# Exploring Synaptic Vesicle Glycoprotein 2A (SV2A) Alterations in Alzheimer's Disease Using Targeted Mass Spectrometry

**DOI:** 10.1002/alz70856_103313

**Published:** 2025-12-25

**Authors:** Henrik Zetterberg

**Affiliations:** ^1^ Institute of Neuroscience and Physiology, Department of Psychiatry and Neurochemistry, The Sahlgrenska Academy at University of Gothenburg, Mölndal, Gothenburg, Sweden

## Abstract

**Background:**

SV2A levels are decreased in Alzheimer's disease (AD) brains, as revealed by Positron Emission Tomography (PET). Whether this decrease is due to synapse loss or SV2A protein loss remains unclear, hindering treatment development and interpretation of SV2A PET as a biomarker for neurodegenerative diseases. To address this, we examined synaptic enriched fractions and crude homogenates from post‐mortem brain tissue.

**Methods:**

Targeted mass spectrometry was performed on individual post‐mortem entorhinal cortex (EC), cerebellum, and centrum semiovale tissue from control (Braak stage 0‐II; *n* = 23), early‐AD (Braak III‐VI; *n* = 21) and AD (Braak V‐VI; *n* = 25) cases. Detergent soluble proteins from total homogenate and synaptoneurosome fractions were digested, desalted and quantified with high flow liquid chromatography and selected reaction monitoring on a 6495 Triple Quadrupole LC/MS system (Agilent Technologies). A panel of 31 synapse associated proteins was measured including SV2A, neurogranin, AP2B1, complexin‐1 and ‐2, synaptophysin, SNAP‐25, syntaxin‐1A and B, β‐synuclein, and NPTX1, NPTX2 and NPTXR. Quality control samples, consisting of homogenate pools, were injected periodically to monitor assay performance.

**Results:**

We found SV2A enriched in the synaptoneurosome fractions compared to total homogenates and lower levels were evident in centrum semiovale as expected. Furthermore, SV2A levels correlated strongly with synaptophysin and previously described interaction partner synaptotagmin‐1 and the SNARE complex proteins. Preliminary analysis showed no significant differences between control, early AD and AD groups in the amount of SV2A protein in the synaptoneurosome fraction, and indicate a trend toward reduction of SV2A protein in AD total homogenates.

**Conclusions:**

These data are consistent with the reduction in SV2A PET signal in AD reflecting loss of synapses rather than loss of protein in remaining synapses. These data form part of the SV2A PET Project, a program of the FNIH Biomarker Consortium which together aim to understand the biological underpinnings of the change in SV2A PET signal in AD.

